# Supplementary material for: MultiCOVID: a multi modal deep learning approach for COVID-19 diagnosis
Source: Sci Rep. 2023 Oct 31;13:18761. doi: 10.1038/s41598-023-46126-8 (PMC10618492; doi:10.1038/s41598-023-46126-8)
Supplement: Supplementary file 1 — Supplementary Information 1. [file 41598_2023_46126_MOESM1_ESM.docx]

**Supplementary**

Image Acquisition, Preprocessing and models

Chest X-Ray (CXR) images were retrieved from the (removed for peer review) Picture Archiving and Communication System (PACS), downloaded in Digital Imaging and Communications (DICOM) format and converted to JPEG using DICOM Toolkit (DCMTK) using a histogram algorithm for image transformation [1]. This conversion strips all the information of the patient. Specifically, we run the command:

$$dcmj2pnm+oj+Pid+Wh3dicom.dcmjpeg.jpeg$$

We processed all images using the same command. Since all images came from the same acquisition procedure, the same value distribution was expected between the different datasets.

All images' modalities obtained were Chest Radiographies (DICOM ID (0008,0060) [CR]) and only PosteriorAnterior (PA) or AnteriorPosterior (AP) views were considered.

For the application of the lung parenchyma segmentation, we first trained a UNet based model with the 758-radiology level segmented images achieving a 95%-pixel accuracy. Then, we applied this model to the image study cohort to obtain predictive masks used for further segmentation. Finally, cropped images were used for training both CXR-only and multimodal deep learning algorithms.

CXR-only models consist on a validated Convolutional Neural Network (CNN) ResNet architecture with 34 layers (resnet-34) [2]. Images were downsampled to 512x512 pixels and augmented using default setting on the fastai API (full list on: <https://eagerai.github.io/fastai/reference/aug_transforms.html>) except for the horizontal flip which is avoided due to laterality preservation. Although mixup augmentation seems to increase model performance [11], we do not used it in our experiments.

Blood-only models are based on TabNet [3] which uses transformer blocks with self-attention [4] in order to select relevant tabular features by using sparsemax function [5]. The data were normalized using Box-Cox transform [7]. Features with high correlation (>95%) to one another were also removed. Model interpretation was performed with SHAP (SHapley Additive exPlanations - https://github.com/lrjball/shap) which assigns each feature an importance value for a particular prediction by using a game theoretic approach [10].

Joint models consist of a multimodal classification task using both CXR and blood test trained end-to-end. In order to prevent overfitting and coordinate the generalization capacity of the single models, we used gradient blending [6]. Specifically, gradient blending is a technique that computes an optimal combination of data sources (modalities) based on their overfitting behaviors. It is used for training multi-modal classification networks, which are networks that take inputs from different sources (such as audio, video, text, etc.) and produce a single output. Gradient blending aims to balance the contribution of each modality to the network’s learning by adjusting their gradients according to their validation losses [6]. MultiCOVID model consists of an ensemble of 5 different Joint models predictions using hard voting to classify the CXR-Blood test pairs. Early stopping was used for model training to define the best model overall in the three models (CXR-only, Blood-only and Joint) independently of the number of epochs performed.

Algorithm Training, Validation, and Testing

CXR-only model weights were initialized using weights trained on the ImageNet dataset, thus images were normalized to the ImageNet mean and standard deviation. The convolutional base of each network was initially frozen, and only the final dense output layer was trained on our clinical dataset. The entire model was then unfrozen, and the model was trained end-to-end on our clinical dataset. Adam optimizer [3] was used together with one cycle policy for superconvergence [4]. Cross entropy loss averaged to fit class imbalance was also used. To calculate the class imbalance, we used a function (‘get_weights()`) that evaluates the number of samples per class and returns the ratio for each category of the training set. A batch size of 8 was used. Early stopping was used to prevent overfitting and to make more comparable the different models.

Blood-only models were built using default parameters in the fastai API. Prior training, hyperparameter tuning was performed using a Bayesian optimization strategy [8] to find the best parameters for training.

Join models were initialized using the best models for both CXR-only models and Blood-only models and finetuned from there. Moreover, Bayesian optimization was also used in order to find the best values for Gradient Blending weights.

Grad-CAM Heatmaps for Prediction exploration

Saliency mapping with gradient class activation maps (Grad-CAM) is a popular method for visualizing feature importance in arriving at a certain prediction in deep learning computer vision classifiers [9]. We applied the Grad-CAM method to individual neural network architectures in the three different datasets (whole, cropped and inverse cropped) to visualize heatmaps of important features for predicting positivity for the different classes.

Hardware and Software Stack

The GPU workstation used for model training and evaluation was a CentOs7 server with 4 Nvidia RTX2080Ti GPUs, running CUDA version 10.0. Model training was performed on fastai Deep Learning API (v2), together with its associated packages for TabNet (<https://github.com/mgrankin/fast_tabnet>) and image interpretation (<https://github.com/Synopsis/amalgam>) and Bayesian Optimization (<https://github.com/fmfn/BayesianOptimization>). All code was written in Python (version 3.7).

Our code base is provided freely on GitHub at [removed for peer review], including weights for each of the individually trained neural network architectures and respective model weights for the weighted ensemble model. Moreover, the anonymized CXR and blood test data is also available upon request.

Variable description

Variables used in the Blood-only models and Joint models for class prediction.

| Variable | Characteristic | Units |
| --- | --- | --- |
| Edad | Age | [Years] |
| Sexe | Sex | n (%) |
| BASFILS | %Basophils | [%] |
| BASFILS_T | Total Basophils | [x10^3/µL] |
| EOSINFILS | %Eosinophils | [%] |
| EOSINFILS_T | Total Eosinophils | [x10^3/µL] |
| H._C._M. | MCH (Mean corpuscular hemoglobin) | [pg] |
| HEMATCRIT | Hematocrit | [%] |
| HEMATIES | Red Blood Cells | [x10^6/µL] |
| HEMOGLOBINA | Hemoglobin | [g/dL] |
| LEUCCITS | Leukocytes | [x10^3/µL] |
| LIMFCITS | %Lymphocytes | [%] |
| LIMFCITS_T | Total Lymphocytes | [x10^3/µL] |
| M._C._H._C. | MCHC (Mean Corpuscular Hemoglobin Concentration) | [g/dL] |
| MONCITS | %Monocytes | [%] |
| MONCITS_T | Total Monocytes | [x10^3/µL] |
| NEUTRFILS | %Neutrophils | [%] |
| NEUTRFILS_T | Total Neutrophils | [x10^3/µL] |
| P-LCR | Platelet large cell ratio | [%] |
| P._D._W. | PDW (Platelet Distribution Width) | [fL] |
| PLAQUETES | Platelets | [x10^3/µL] |
| RDW-CV | Red cell distribution width coefficient of variation | [%] |
| RDW-SD | Red cell distribution width standard deviation | [fL] |
| V._C._M. | MCV (Mean Corpuscular Value) | [fL] |
| V._P._M. | MPV (Mean platelet Volume) | [fL] |

1. Muschelli J. “dcmtk: Wrapper for 'DICOM' Toolkit ('DCMTK')” (https://github.com/muschellij2/dcmtk)
2. He K., Zhang X., Ren S., Sun J., "Deep Residual Learning for Image Recognition," 2016 IEEE Conference on Computer Vision and Pattern Recognition (CVPR), 2016, pp. 770-778, doi: 10.1109/CVPR.2016.90.
3. Arik, S. Ö. and Pfister, T. (2021) “TabNet: Attentive Interpretable Tabular Learning”, Proceedings of the AAAI Conference on Artificial Intelligence, 35(8), pp. 6679-6687. Available at: https://ojs.aaai.org/index.php/AAAI/article/view/16826 (Accessed: 14October2021).
4. Vaswani, A., Shazeer, N.M., Parmar, N., Uszkoreit, J., Jones, L., Gomez, A.N., Kaiser, L., & Polosukhin, I. (2017). Attention is All you Need. ArXiv, abs/1706.03762.
5. Martins A., Astudillo R. 2016. From softmax to sparsemax: a sparse model of attention and multi-label classification. In Proceedings of the 33rd International Conference on International Conference on Machine Learning - Volume 48 (ICML'16). JMLR.org, 1614–1623.
6. Wang, W., Tran, D., & Feiszli, M. (2019). What Makes Training Multi-Modal Networks Hard? ArXiv, abs/1905.12681. (Accessed: 14October2021).
7. Box, G. E. P., and D. R. Cox. “An Analysis of Transformations.” Journal of the Royal Statistical Society. Series B (Methodological), vol. 26, no. 2, [Royal Statistical Society, Wiley], 1964, pp. 211–52, http://www.jstor.org/stable/2984418.
8. Snoek, J., Larochelle, H., & Adams, R.P. (2012). Practical Bayesian Optimization of Machine Learning Algorithms. NIPS.
9. R. R. Selvaraju, M. Cogswell, A. Das, R. Vedantam, D. Parikh and D. Batra, "Grad-CAM: Visual Explanations from Deep Networks via Gradient-Based Localization," 2017 IEEE International Conference on Computer Vision (ICCV), 2017, pp. 618-626, doi: 10.1109/ICCV.2017.74.
10. Lundberg, Scott M. and Su-In Lee. “A Unified Approach to Interpreting Model Predictions.” NIPS (2017).
11. Nishio M, Noguchi S, Matsuo H, Murakami T. Automatic classification between COVID-19 pneumonia, non-COVID-19 pneumonia, and the healthy on chest X-ray image: combination of data augmentation methods. Sci Rep. 2020;10(1):17532. Published 2020 Oct 16. doi:10.1038/s41598-020-74539-2
